# Entacapone Reduces Cortical Activation in Parkinson's Disease with Wearing-Off: A *f*-MRI Study

**DOI:** 10.1371/journal.pone.0096806

**Published:** 2014-05-15

**Authors:** Nicola Tambasco, Marco Muti, Pietro Chiarini, Roberto Tarducci, Stefano Caproni, Anna Castrioto, Pasquale Nigro, Lucilla Parnetti, Pietro Floridi, Aroldo Rossi, Paolo Calabresi

**Affiliations:** 1 Clinica Neurologica, Azienda Ospedaliera-Università di Perugia, Perugia, Italy; 2 Servizio di Fisica Sanitaria, Azienda Ospedaliera di Terni, Terni, Italy; 3 Servizio di Neuroradiologia, Azienda Ospedaliera di Perugia, Perugia, Italy; 4 Servizio di Fisica Sanitaria, Azienda Ospedaliera di Perugia, Perugia, Italy; 5 I.R.C.C.S. – Fondazione S.Lucia, Roma, Italy; Philadelphia VA Medical Center, United States of America

## Abstract

**Background and Purpose:**

Wearing-off is one of the most frequent problems encountered by levodopa-treated patients. Entacapone, a peripheral inhibitor of catechol-O-methyltransferase (COMT), reduces this motor complication by prolonging the effect of levodopa. We sought to understand the impact of COMT-inhibition on movement execution in PD patients with wearing-off by comparing functional magnetic resonance imaging (*f*-MRI) activation patterns prior to and during entacapone treatment. Our hypothesis was to determine whether changes in cortical activation are associated to COMT-inhibitor treatment.

**Methods:**

Nine levodopa-treated non-demented PD patients with wearing-off were prospectively studied in two f-MRI session, prior to and during entacapone treatment. A group of control subjects were also studied for comparison.

**Results:**

The patients significantly improved under COMT-inhibitor treatment based on home diaries. *F*-MRI results showed that at baseline the patients presented a bilateral activation of the primary motor, controlateral premotor cortex and supplementary motor area, as well as ipsilateral cerebellum. During treatment with entacapone, PD patients showed reductions in the activations of these cortical areas and a decreased activation in the ipsilateral cerebellum.

**Conclusions:**

Our preliminary findings indicate that *f*-MRI is able to detect cortical activation changes during long-term modulation of dopaminergic treatment in PD patients with wearing-off, and thus, this technique could be further investigated in advanced PD patients.

## Introduction

The most frequent complication during levodopa treatment in Parkinson's disease (PD) patients is a progressive shortening in the duration of levodopa action [1]. The corresponding effect is a predictable “wearing-off” (WO), where the symptomatic benefit from a given dose of levodopa is not maintained until the next scheduled dose takes effect [2]. Thus, strategies to prolong dopaminergic stimulation, such as the combination of levodopa with a peripheral inhibitor of the enzyme catechol-*O*-methyltransferase (COMT), are required [3]. Although long-term changes in striatal dopamine receptors have been observed, the mechanisms contributing to the emergence of these motor complications are not yet fully understood [4]. Despite its clinical efficacy, the impact of levodopa on the motor cortex has been investigated by the blood-oxygen-level-dependent (BOLD) technique with heterogeneous results. This variability is likely related to the selection of different parameters, such as the type of motor tasks (random or sequential, self-paced or external-guided, simple or complex), disease severity and effects of therapy. The influence of therapy on cortical activation in PD has primarily been studied with levodopa. For example, acute levodopa intake, has been shown to increase activation in the bilateral supplementary motor area (SMA) and the contralateral primary motor cortex (M1) in *de novo* patients [5], whereas in chronically levodopa-treated PD, levodopa has been shown to decrease the bilateral activation of M1 and lateral premotor cortex (LPM) and to increase activation in the SMA [6]. Compared with levodopa alone, COMT-inhibitors allow for more continuous dopaminergic stimulation, thereby obtaining a reduction in fluctuations [7]. To fully understand the impact of COMT-inhibition on cortical motor areas in PD patients with WO, we compared functional magnetic resonance imaging (*f*-MRI) activation patterns in PD patients prior to and during entacapone treatment.

## Experimental Procedures

### 1. Subjects

Nine right-handed patients with advanced PD, fulfilling the UKPDBB criteria [8] (mean age: 67.0±5; males: 4; disease duration: 5.4 years; mean UPDRS (part III) in ON: 17.0, in OFF-MED 30.1, in ON-MED plus Entacapone: 16.2; levodopa (mg/d): 461.1±151; mean MMSE: 27.2) were included (Table 1). The response to acute challenge with levodopa or apomorphine and WO, as recorded by home diary entry, were evaluated for PD diagnosis. *Inclusion criteria* : age >40 and <75 years, disease duration >5 years, UPDRS III-motor part score ≥18 in *off* condition, Hoehn and Yahr scale ≥2, MMSE score >24, WO phenomenon, levodopa treatment. *Exclusion criteria* : dementia and depression/psychiatric illness (according to DSM IV criteria), all secondary causes of parkinsonism, epilepsy, history of other neurological disorders, stroke and/or brain trauma and drug or alcohol abuse.

**Table 1:** Baseline characteristics of PD patients.

	Age	Gender	Disease duration (yr)	MMSE	most affected side	Mean UPDRS (part III) On Med	Mean UPDRS (part III) Off Med	Mean UPDRS (part III) On Med plus Entacapone	LDopa (mean mg/d)	Entacapone (mg/d)
1	73	m	5	28	R	18	32	18	450	600
2	62	f	6	30	L	20	32	18	600	800
3	62	f	5	28	R	12	27	12	400	800
4	71	m	5	29	R	18	31	15	300	600
5	70	f	6	28	R	19	29	18	350	600
6	71	m	6	25	R	22	32	20	400	600
7	61	m	6	25	L	10	28	11	450	600
8	62	f	5	25	R	22	30	22	800	800
9	72	f	5	27	R	12	36	12	400	800

Neither modification of prescribed medications nor the use of dopamine agonists was allowed during the study. The control group was composed of ten age- and sex-matched subjects (mean age: 65, 7±6; males: 6; Mean MMSE: 28.3). Written informed consent was obtained from all patients and controls. To assess the capacity to consent of the subjects, baseline cognitive status was determined by neurological assessment and consensus diagnosis for dementia using the Diagnostic and Statistical Manual of Mental Disorders [9]. The study has been performed in accordance with the Declaration of Helsinki. The local ethics committee (CEAS Umbria) approved this study.

### 2. Functional MRI Acquisitions

A 1.5 T magnet (Signa Advanced GE/Standard) with field homogeneity optimized by auto shimming was utilized. Anatomical correlation of the activation maps was provided by a Spin echo sequence (TR/TE 500/20 ms) performed in the same axial plane as the activation maps. *F*-MRI exams were preceded by a clinical acquisition that was observed by a neuroradiologist who noted the normality of the radiological findings.

Activation maps were acquired with an SPGR sequence (TR = 640 ms, TE = 48 ms, flip angle 17^°^, FOV 22×16 cm, matrix  = 256×128) and 10 contiguous slices were used for the study (thickness  = 6 mm, 1 excitation, total acquisition time  = 19 seconds), 3 of which were centered on the adjacent motor areas highlighting the primary motor cortex (M1), the secondary motor cortex (LPM and SMA) and the cerebellum. Every axis of each scan was checked for possible movement of the subject. Functional data were acquired at rest and during finger movements. Patients underwent *f*-MRI twice: prior to and 30–45 days after 200 mg entacapone was added to each levodopa dose. Each exam was acquired at approximately the same hour of day to minimize pharmacological variability. Furthermore, the timing for Levodopa assumption varies from 3 to 4 times per day and all the exams were performed from 3 to 4 hours after Levodopa or Levodopa/Entacapone administration for all the subjects.

### 3. Motor Task

The activation task used for this study has already been described [10]. The subject was invited to perform a self-paced sequential movement with the right hand and executed with a sequential and rhythmic movement, touching the thumb to the 2nd, 3rd, 4th and 5th fingers, in seven blocks. Each block was characterized by an acquisition time of thirty seconds followed by thirty seconds of rest. This movement was used for the activation of M1, LPM, SMA and cerebellum. All subjects were instructed to practice this sequential task right before the scan until they were able to perform the full series without errors, at a frequency of approximately 1 Hz with an intermediate amplitude. An auditory pacing signal was delivered to begin the motor task and to return to the resting state. No external cue was given to help the subjects move at the specified rate.

### 4. Statistics

Statistical analysis was performed utilizing SPM2 software (Wellcome Department of Imaging Neuroscience, Institute of Neurology, University College London, UK). Basing on previous evidences in literature about motor network, for the task execution by both hands bilateral M1, SMA, controlateral LPM and ipsilateral cerebellum were considered as regions-of-interest (ROIs) [11].

The functional images were co-registered and realigned to the first volume to correct for head translation or rotation during the scanning and to avoid incorrect spatial coordinates of activated voxels, as well as normalised to the stereotaxic space of Talairach and Tournoux [12] using the three-dimensional volume [13]. The images were also spatially smoothed with a Gaussian kernel of 8 mm full-width half maximum and temporally smoothed with a Gaussian kernel (FWHM  = 8 s) [13].

Statistical analysis of the activation obtained during task performance was based upon an epoch-related experimental design. The data obtained were modelled with a hemodynamic response function, with impulsive local flux variation. The sum of hemodynamic variations during an active period allowed for the calculation of mean cortical activation during an exercise performance. A General Linear Model, y = (β/βerr)*x+c [13] was applied to obtain, for activation of each of the five ROIs, the corresponding T-score. This score reflects its activation size (cluster of voxels, k) [14], [15]. This process was applied to all subjects at baseline and to PD patients 30–45 days after the add-on of 200 mg entacapone to each levodopa dose. Then, a two-way T-test was performed to compare the activation of each ROIs between patients and controls before and after the treatment.

## Results

Seven out of nine PD patients and all the controls completed the trial. Two PD patients were excluded: one for poor tolerance to entacapone and one for the insufficient technical quality of the exam. The mean duration of the treatment with entacapone before the second *f*-MRI was 37.8±4 days. The mean dose of entacapone per day was 688.9 mg.

### 1. Clinical Results

The patient group showed a significant response to the COMT-inhibitor. The ON time, based on home diaries, significantly improved (number of hours in OFF: baseline 8.5; last visit at 1 month 6.4; p<0.01). The hand movements of the PD patients had the same frequency in both section of the MRI exam (26±2.1/min-first exam and 26±4.3/min-second exam). None of the patients were dyskinetic at first evaluation nor have been experienced dyskinesias during the study.

### 2. Functional MRI Results

Motor areas were analyzed prior to and during entacapone therapy. At the baseline condition, in PD patient group, the activation of bilateral M1, left LPM, SMA and right cerebellum was observed. At the same time, in control group, the activation of left M1, left LPM, SMA and right cerebellum was detected. The activations of all the ROIs resulted statistically different between PD and control group (p<0.05) (Table 2). After the add-on of the entacapone therapy, PD patients showed the activation of left M1, left LPM, SMA and right cerebellum. The activations of all the ROIs resulted statistically different form PD group at baseline (p<0.05), while did not significantly differ from control group (p>0.05) (Table 2).

**Table 2:** Group analysis for right finger tapping: site of maximum activation for each ROI in controls and in patients before and during entacapone treatment.

Region	Controls				PD patients (baseline)*				PD patients (during entacapone)#°			
	x	y	z	T score	x	y	z	T score	x	y	z	T score
**SMA**	8	−12	64	6.9	−5	−4	60	7.9	8	−2	65	7.2
**Left M1**	−26	−28	56	8.5	−54	−22	44	9.4	−50	−18	46	8.5
**Right M1**	-	-	-	-	44	−26	36	7.9	-	-	-	-
**Left LPM**	−30	−11	59	7.2	−34	−16	70	7.6	−34	−14	68	7.2
**Right Cerebellum**	10	−56	−16	8.2	26	−52	−28	8.8	14	−58	−18	8.4

Talairach x,y,z coordinates and peak T-scores are shown. SMA, supplementary motor area; M1, primary motor cortex; LPM, lateral premotor cortex.

*for each ROI, the 2-way T-test resulted statistically different between PD patients at baseline and controls (p<0.05).

# for each ROI, the 2-way T-test did not result statistically different between PD patients during entacapone and controls (p>0.05).

°for each ROI, the 2-way T-test resulted statistically different between PD patients during entacapone and controls (p<0.05).

## Discussion

The results of this study show that, in PD patients with WO, COMT-inhibition is associated with BOLD signal modifications. The BOLD changes are characterized by a decreased activation of the bilateral M1 and contralateral LPM and SMA, as well as decrease activation in the ipsilateral cerebellum, during the execution of motor task with both hands.

The motor cortex has been the region most studied by *f*-MRI to understand the relationship between movement and brain activations. Several previous studies showed that acute dopaminergic stimulation exerted different cortical motor changes depending on the clinical conditions. In *de novo* PD, levodopa intake induces an increase in motor cortex activation in response to simple hand movements [5]. In contrast, in treated patients, acute levodopa induces a reduction in activation of motor areas [6]. Levodopa also leads to changes in the frontal area in response to cognitive stimuli [16]. Dopaminergic therapy has a specific dose-dependent effect on both the default mode network integrity and task-related brain activations in cognitively intact PD patients [17].

Thus, the frontal cortex depends on dopamine to respond to voluntary movements, and our study strengthens this notion by the observation of reduced activation of bilateral M1 and contralateral LPM and SMA, even in response to the mild increase induced by a COMT-inhibitor. In fact, in advanced PD, the progressive degeneration of nigrostriatal dopaminergic terminals could contribute to the pathogenesis of WO *via* pharmacokinetics and pharmacodynamics mechanisms [4]. In these patients, rapid changes in the synaptic levels of dopamine after levodopa administration have been observed [1]. This suggests that fluctuating patients may be characterized by more severe nigrostriatal damage [18]. In this scenario, an increased dopamine turnover might play a relevant role in levodopa-related WO [4]. In fact, the addition of entacapone causes an increase in levodopa bioavailability leading to an increase in dopamine concentration [19] and, ultimately, a more continuous stimulation. This effect is clinically relevant inducing an amelioration of disability evaluated by rating scale as UPDRS II and UPDRS III or ADL in PD patients with WO [20]. Levodopa, which directly acts on the pathways modulating the functional striato-cortical connectivity, generally leads to more efficient performance [21]. While dopamine deficiency leads to abnormalities in corticostriatal interaction during frontal lobe tasks [22], dopamine replacement tends to increase functional connectivity between the striatum and the prefrontal cortex normalizing cortical activation [23]. Furthermore, functional reorganization of the primary motor cortex may occur after prolonged dopamine treatment. This is consistent with the view that the motor cortex is overactive in PD patients to compensate for the deficient basal ganglia function [24].

Increasing attention has been dedicated over recent years to the *f*-MRI study of other structures that integrate with cortical areas [25]. The BOLD technique has contributed to the understanding of the two main pathways to cortical areas: the basal ganglia- thalamo-premotor loop to the SMA [26] and the cerebellar-parieto-lateral premotor loop to the mesial premotor/prefrontal circuit [6]. In our study, the ipsilateral cerebellar hemisphere showed an increase in baseline conditions compared with the controls and a reduction during treatment with entacapone. This is consistent with other studies that demonstrated an increase in cerebellar activity in *off* state. The role of the cerebellum has been demostrated to be in opposition to the putamen and is hyperactive in the off-medication and reduces activation when the patients is turned on [27]. A proposed explanation for the basal ganglia hypoactivation and cerebellum hyperactivation is that there is a mechanism of reversal compensation of the cerebellum when the basal ganglia were hyperactive [28].

This study has a number of limitations. First, the small number of subjects allowed only a limited analysis. Second, the simple design used in our preliminary report was aimed to investigate the impact of COMT-inhibition on movement execution in PD patients with WO during a self-generated movement on motor areas and, then, could be further completed by a cross-over study. Third, the short duration of the *f*-MRI acquisition and the motor task were designed to make the study feasible in patients who otherwise would not have been able to accomplish the task. Nevertheless, our study demonstrates for the first time that long-term modulation of dopaminergic transmission by COMT inhibition is able to affect cortical activation in patients suffering from advanced PD. Further studies involving a larger cohort of patients are required to validate this hypothesis.
